# Associations Between Self-Stigma and Health Promotion Behaviors in Overweight/Obese Patients With Type 2 Diabetes: A Cross-Sectional Study

**DOI:** 10.1155/jdr/9142949

**Published:** 2025-05-26

**Authors:** Shilian Niu, Rao Li, Li Yuan, Dan Huang

**Affiliations:** Department of Endocrinology and Metabolism, West China Hospital, Sichuan University/West China School of Nursing, Sichuan University, Chengdu, China

## Abstract

**Background:** Patients experiencing stigma are more prone to engage in unhealthy behaviors. The correlation between stigma and health-promoting behaviors in overweight/obese T2DM patients is unclear. Therefore, this study aimed to investigate the association between the two in this particular population.

**Methods:** This cross-sectional study, conducted from July 2022 to July 2023 at the Department of Endocrinology and Metabolism of a tertiary general hospital in Chengdu, Sichuan Province, China, used convenience sampling to enroll overweight/obese T2DM patients. A structured questionnaire was used to obtain information on sociodemographic and clinical characteristics. Respondents were assessed for stigma and health-promoting behaviors using the SSCI and T2DHPS scales. Data were analyzed using SPSS version 27.0, with a significance level of *p* < 0.05.

**Results:** The majority was males (64%). The average BMI of patients was 27.27 ± 3.11 (kg/m^2^), and the average HbA1c was 8.36 ± 1.81 (mmol/mol). The overall stigma score of overweight/obese T2DM patients was 4.19 ± 8.69, and the overall health-promoting behaviors score was 87.75 ± 16.75. Pearson correlation analysis revealed a significant correlation between stigma and the overall score of health promotion behaviors (*r* = −0.144, *p* < 0.05). The multiple linear regression analysis showed that the hypoglycemia incidence, patient origin, GLP-1RA use, and stress management explained 23.9% of the patients' feelings of stigma.

**Conclusions:** This study found that there was a correlation between the level of stigma and health-promoting behaviors in overweight/obese patients with T2DM. Hypoglycemia, inpatient, GLP-1RA treatment, and stress management significantly predicted stigma among overweight/obese patients. These findings have implications for healthcare providers, as they can develop intervention strategies by assessing the levels of stigma and stress management in overweight/obese patients with T2DM, to help reduce stigma and promote healthy behaviors among these patients.

## 1. Introduction

Stigma has multiple negative impacts on an individual's dignity, health, self-care, and well-being [1–3]. International organizations have called on drawing more attention to stigma, aiming to address stigma in disease management and make it a research priority. Stigma is primarily caused by blame, fear, and disgust resulting from negative stereotypes and misinformation [4]. It is estimated that by 2040, the number of obese adults will increase by sixfold and the prevalence of diabetes will increase to 642 million [5]. Due to the rapid urbanization and the change of lifestyle, the rate of overweight/obesity and diabetes may continue increasing. This group is prone to have feelings of shame and low self-esteem due to being overweight, changes in appearance, or recognition of their diseases [6]. With the awareness and publicity of diabetes and overweight/obesity through traditional concepts and new social media, this group has been labeled as gluttonous, lazy, and irresponsible. Patients are prone to feel stigma and even aggravate this psychological burden [7]. Studies have shown that patients experiencing stigma are more prone to engage in unhealthy behaviors such as overeating and eating to reduce stress, higher levels of diabetes distress [8], poorer self-management of diabetes, and fewer clinical visits, leading to underdiagnosis and undertreatment of the disease [9]. Diabetes self-care strategies emphasize a series of complex and rigorous disease management activities such as adjusting diet, exercise, adherence of medication regimens, and blood glucose self-monitoring. Patients frequently encounter emotional distress and diminished quality of life during disease management. Health-promoting behaviors have been recognized as important strategies for preserving and enhancing quality of life in individuals with chronic conditions, with a focus on sustaining proactive engagement across life domains [10, 11]. Health-promoting behaviors help alleviate and reduce the risk of microvascular and macrovascular complications in individuals with high body mass index (BMI) and diabetes and improve the individual's positive psychological level, to improve the quality of life [12]. Previous research has found that experiencing stigma can negatively impact patients' health behaviors, such as affecting the emotional state of people with diabetes. However, there is a lack of relevant research on whether stigma has an impact on health-promoting behaviors in overweight/obese patients with Type 2 diabetes mellitus (T2DM) [13]. Therefore, this study aimed to investigate the relationship between stigma and diabetes health-promoting behaviors in overweight/obese patients with T2DM in a tertiary general hospital in Sichuan Province, China.

## 2. Methods

### 2.1. Study Design

This study was cross-sectional. All patients were recruited from West China Hospital, which is a 4900-bed tertiary teaching hospital affiliated with Sichuan University in Chengdu and a leading medical center in West China. Ethical approval was obtained from the Ethical Research Committee of Sichuan University West China Hospital (approval number: 2022 Review [1500]).

### 2.2. Setting

The study was conducted at West China Hospital of Sichuan University from July 2022 to July 2023, with standardized protocols implemented to ensure methodological rigor. All participants were fully informed of the study's purpose and significance, voluntarily completed the questionnaire survey, and provided written informed consent. Inclusion criteria include (a) age ≥ 18 years, (b) fulfillment of the 1999 WHO diagnostic criteria for diabetes mellitus, and (c) BMI ≥ 25 kg/m^2^. Exclusion criteria comprised severe psychiatric disorders, cognitive impairment, absolute bedridden, or disturbance of consciousness. Before commencement of the survey, investigators underwent professional training to ensure comprehensive understanding of research objectives and data collection methods. All investigators were qualified medical personnel. When participants had difficulty completing the questionnaire independently, the investigator used neutral language to assist and accurately record responses. Missing data were promptly addressed through communication with patients.

### 2.3. Sample

Participants were recruited through convenience sampling from both inpatient wards and outpatient clinics of the Department of Endocrinology and Metabolism at the hospital. Given the inclusion of 25 variables in the questionnaire, a sample size of at least 138 was deemed necessary, following Kendall's rough estimation method, which suggested a sample size of 5–10 times the number of variables. Consequently, 200 questionnaires were distributed, resulting in a valid return rate of 98.5%, with 197 questionnaires being considered valid for analysis.

### 2.4. Data Collection and Variables

#### 2.4.1. Sociodemographic and Clinical Characteristics

General demographic characteristics obtained included gender, age, education, marital status, occupation, per capita monthly household income, and patient origin. Clinical information of the participants was also collected, such as BMI, time since diagnosis (in years), treatment modalities, diabetes-related complications, comorbidities of chronic diseases, abdominal obesity, glycated hemoglobin (HbA1c) levels, and the use of GLP-1RA therapy. BMI was calculated as the ratio of body weight in kilograms to squared body height in meters (kg/m^2^). Participants were categorized as overweight (BMI, 25.0–29.9 kg·m^2^), or obese (BMI ≥ 30.0 kg·m^2^), according to the criteria of the WHO International BMI Classification of adults [14]. The number of diabetes-related complications was calculated as a simple composite of six complications, referencing the diabetes complications index (DCI), with scores ranging from 0 to 6 [15]. The number of comorbidities of chronic diseases was calculated by a simple composite of comorbidity types outlined in the International Classification of Diseases (ICD-10) overview [16]. Abdominal obesity was determined by waist perimeter (WC). WC was measured midway between the bottom of the twelfth rib and the anterior superior iliac spines while the subject was standing. Abdominal obesity was defined as having waist circumference > 90 cm in men and > 85 cm in women [17]. HbA1c levels were based on the most recent laboratory test results within the past 3 months for each patient.

#### 2.4.2. Stigma

Stigma was assessed using the Stigma Scale for Chronic Illnesses (SSCI). The validated Chinese version of the SSCI comprises 24 items evaluating both internalized and external stigma. The first 13 items measure internalized stigma by inquiring about participants' self-perceived stigmatization, while the remaining 11 items assess external stigma through perceived stigmatizing behaviors from others. The total scale demonstrated excellent reliability with a Cronbach's alpha coefficient of 0.951, having been previously validated in chronic disease populations, including diabetes. Responses were recorded on a 5-point Likert scale [18] with anchors ranging from “Never” (1 point) to “Always” (5 points). The total score ranges continuously from 24 to 120, with higher scores indicating greater self-stigmatization. In the current study, the scale showed high internal consistency (Cronbach's alpha = 0.948).

#### 2.4.3. Health-Promoting Behaviors

Health-promoting behaviors were assessed using the Type 2 Diabetes Health Promotion Scale (T2DHPS). The T2DHPS consists of 28 items across six dimensions: physical activity (7 items), risk reduction (7 items), stress management (5 items), enjoy life (3 items), health responsibility (3 items), and healthy diet (3 items). This scale was developed through adaptation and modification of the Health-Promoting Lifestyle Profile II (HPLP-II), with subsequent cultural adaptation to the Chinese context by mainland scholars [19]. The T2DHPS demonstrates specificity for T2DM populations and has established satisfactory reliability and validity. Responses were measured using a 5-point Likert scale (*Never* = 1, *Occasionally* = 2, *About half the time* = 3, *Often* = 4, and *Always* = 5). The total score ranges from 28 to 140, with higher scores indicating stronger diabetes-related health promotion behaviors. In the current study, the T2DHPS demonstrated high reliability and validity, achieving a Cronbach's alpha coefficient of 0.905.

### 2.5. Statistical Analysis

SPSS version 27.0 was used for statistical analyses. Categorical variables were described by frequencies and percentages, and continuous variables were described by means and standard deviations. *T*-tests and ANOVA were employed to assess variations in stigma across sociodemographic variables. Pearson correlation analysis was utilized to examine the association between stigma and health promotion behaviors among overweight/obese diabetic patients. Multiple linear regression analysis was used to explore the predictors of stigma. *P* values of less than 0.05 were considered statistically significant.

## 3. Results

This study enrolled 200 overweight/obese individuals diagnosed with T2DM, recruited from both outpatient and inpatient settings. Written informed consent was obtained from all participants. However, three responses were considered incomplete or inconclusive and were excluded. The analysis was based on data from 197 valid responses.

Table 1 shows the demographic characteristics of the study subjects and the univariate analysis of stigma with different characteristics. Among the 197 overweight/obese patients with T2DM, 126 (64%) were males and 71 (36%) were females. The age of the patients range from 18 to 84, with an average age of 54.12 ± 13.14 years. The average BMI of patients was 27.27 ± 3.11, 133 (67.5%) patients were overweight, 64 (32.5%) patients were obese, and the average HbA1c was 8.36 ± 1.81 (mmol/mol). The average number of diabetes-related complications was 0.58 ± 0.93, and the average of chronic disease comorbidities was 1.58 ± 1.15. The results of the *T*-test and ANOVA showed statistically significant differences between patients in terms of monthly household income, diabetes complications, hypoglycemia, patient origin, and GLP-1RA treatment (*p* < 0.05). LSD post hoc test was conducted for more than three groups of items, *p* < 0.05. The LSD post hoc test results showed that there was a significant difference between the group with a monthly household income of > 10,000 RMB and the other groups.

The overall stigma score of overweight/obese T2DM patients was 4.19 ± 8.69, the overall health-promoting behaviors score was 87.75 ± 16.75 (see Table 2 for details).

Table 3 presents the results of an analysis examining the correlation between stigma and health-promoting behaviors. Pearson correlation analysis revealed a significant correlation between stigma and the overall score of health promotion behaviors (*r* = −0.144, *p* < 0.05). However, only two dimensions, namely, stress management (*r* = −0.312, *p* < 0.01) and enjoy life (*r* = −0.254, *p* < 0.01), exhibited a significant negative correlation with self-stigma.

In the multiple regression analyses, a stepwise approach was used to select the independent variables. Statistically significant sociodemographic data were then modeled, including monthly household income, hypoglycemia incidence, patient origin, GLP-1RA use, diabetes complications, and stress management. Regression analyses were performed on dummy variables for each group. The results of the analyses showed that hypoglycemia, patient origin, GLP-1RA use, and stress management were the main predictors of stigma in overweight/obese T2DM patients (*F* = 7.162, *p* < 0.001, *R*^2^ = 0.278, *R*^2^_ad_ = 0.239) (Table 4).

The results of the multiple linear regression analysis showed that the presence of hypoglycemia incidence, patient origin, GLP-1RA use, and stress management explained 23.9% of the patients' feelings of stigma. Stigma was higher in thosesusceptibility to hypoglycemia, inpatients, GLP-1RA use, and weak stress management.

## 4. Discussion

This study aimed to examine the association between stigma and health promotion behaviors among overweight/obese T2DM patients in China. In this study, the overall stigma scores measured 54.19 ± 8.69, exceeding values reported by Li et al. in their investigation of Chinese T2DM patients with normal BMI status [20]. The distinctive feature of this study group lies in the fact that the overweight/obese cohort frequently encounters socially imposed negative perceptions, such as those related to body size, rendering them susceptible to external discrimination, thereby eliciting negative emotions [21]. The average score for health promotion behaviors (87.75 ± 16.75) exceeded those reported by Wang et al. in a study conducted in China, potentially due to differences in age distribution among the study cohorts [22]. Additionally, the research revealed a correlation between stigma and reduced engagement in health promotion behaviors among patients, particularly evident in decreased stress management and enjoy life. In conclusion, these preliminary findings suggested the necessity for further research to elucidate the mechanisms by which stigma influences health behaviors within this population.

This study found that patients with a history of hypoglycemia, hospitalization, use of GLP-1RA therapy, and poorer stress management skills face more pronounced stigma. Particularly among overweight/obese T2DM patients, those with a history of hypoglycemia also exhibit heightened stigma, aligning with prior research suggesting that fear of hypoglycemia exacerbates stigma as patients avoid public situations to prevent hypoglycemic episodes and gradually withdraw from social relationships [20].

The elevated stigma levels observed in inpatients compared to outpatients corroborate existing evidence that T2DM patients face heightened stigmatization risks from healthcare providers, potentially exacerbating increased health risks as a result of prejudicial attitudes and negative stereotypes of lifestyles about weight and diabetes [23]. Inpatients experience higher levels of shame than outpatients which supports previous research suggesting that patients may face greater stigma from healthcare providers due to discriminatory attitudes and negative stereotypes surrounding weight and diabetes management. This heightened sense of stigma could lead to increasing health risks for patients, which could even be exponential. The finding that patients treated with GLP-1RA were more likely to experience stigma was unexpected. Other previous studies have suggested a role for treatment in increasing stigma. The patients in this study who used GLP-1 RA medication may have seen their use of the medication as a ‘failure' in their ability to achieve weight control through their behavior and lifestyle. This societal bias may have led to negative feelings about themselves and increased stigma. Patients with poor stress management skills have higher levels of stigma. Previous studies have shown that most people with diabetes report higher levels of perceived stress, anxiety, and fatigue than people in the general population [24, 25]. Stigma can diminish patients' ability to cope with stress [9]. Stress management interventions in HIV populations have effectively reduced stigma [26, 27]. However, intervention studies targeting diabetes patients are still lacking at present.

Our study revealed a significant negative correlation between two dimensions of diabetes health promotion behaviors—stress management and enjoy life—and stigma. This finding aligns with previous research on perceived stress among diabetic populations. It may be attributed to the significant increase in the risk of complications associated with chronic disease, coupled with the burden of healthcare expenditure, treatment, societal judgment, and self-blame, all of which can profoundly impact the emotional well-being of patients [28–30]. Hence, there is a critical need to develop individualized psychosocial support programs aimed at fostering positive changes in stress management skills and stigma reduce. Physical activity, risk reduction, health responsibility, and healthy eating were not clearly shown to correlate with stigma in this study. However, Puhl et al. [9], Cho et al. [31], Khezerloo and Feizi [32] showed that diabetes stigma was associated with poorer diabetes self-management. This may be because diabetes requires long-term management in terms of diet, exercise, medication, and blood glucose monitoring, and patients consider these behaviors as part of their lives, so physical activity, risk reduction, health responsibility, and healthy eating scored higher. Therefore, this study suggests that healthcare professionals should pay more attention to patient's psychological status, implement targeted interventions to mitigate the adverse effects of stressful life events, enhance psychological adaptability, reduce patients' sense of stigma, and ultimately elevate quality of life. However, the sources of participants' perceived stress, which may influence stigma, were not explored in detail in this study.

Furthermore, evidence indicates that adults with T2DM exhibit nearly double the prevalence of depression compared to the general population [33]. While stigma elevates depression risk, depression itself appears to more directly impair treatment adherence. The attenuated negative association between stigma and health-promoting behaviors observed in this study may be explained by unmeasured mediating variables (e.g., depression severity) that attenuated the direct association. However, there is currently a paucity of relevant research, and further investigations are warranted to elucidate these effects.

This study has several limitations that warrant acknowledgment. Firstly, its cross-sectional design implies the necessity for follow-up investigations with larger sample sizes to validate the impact of stigma on health-promoting behaviors, such as dietary habits and exercise, among overweight/obese diabetic patients. Secondly, the recruitment of all participants from the specialized inpatient/outpatient department of a general hospital underscores the need for broader representation and expansion in both the size and source of the study population. Moreover, the stigma measurement tool utilized in this study did not rigorously distinguish weight stigma from diabetes stigma, limiting its capacity to accurately discern whether patients' perceived stigma stemmed from weight-related issues or their diabetes diagnosis.

## 5. Conclusion

This study found that there was a correlation between the level of stigma and health-promoting behaviors in overweight/obese patients with T2DM. Healthcare providers should adopt integrated approaches addressing stigma through psychological support, family engagement, and stigma-sensitive communication to mitigate adverse outcomes. Future research should explore whether there are mediating factors between stigma and health behaviors, distinguish between weight stigma and diabetes stigma, and develop targeted interventions to reduce stigma.

## Figures and Tables

**Table 1:** Distribution of stigma scores in overweight/obese patients with T2DM with different demographic characteristics (*N* = 197, *x* ± *s*).

**Characteristic**	**N** ** (%)**	**Stigma**	**t**/**F**	**p**
Sex			3.630	0.058
Male	126 (64.00)	53.25 ± 7.93		
Female	71 (36.00)	55.85 ± 9.74		
Age			1.627	0.199
< 40	29 (14.70)	52.03 ± 1.12		
40–60	95 (48.20)	55.19 ± 0.97		
> 60	73 (37.10)	53.74 ± 0.99		
BMI			0.314	0.576
Overweight	133 (67.50)	53.8 ± 8.90		
Obese	64 (32.50)	54.98 ± 8.26		
Occupation			0.116	0.891
Employed	100 (50.80)	54.11 ± 8.55		
Retired	91 (46.20)	54.37 ± 8.95		
Others	6 (3.00)	52.67 ± 8.33		
Marriage			1.038	0.356
Married	9 (4.60)	56.56 ± 7.38		
Unmarried	181 (91.90)	53.93 ± 8.58		
Divorced or widowed	7 (3.60)	57.86 ± 12.66		
Educational			1.402	0.243
Primary school and below	32 (16.50)	55.19 ± 8.64		
Junior high school	43 (22.60)	56.05 ± 10.45		
High school/college	31 (15.70)	54.16 ± 10.43		
Undergraduate and above	91 (45.20)	54.25 ± 8.06		
Residential status			1.906	0.130
Living alone	17 (8.60)	56.35 ± 9.50		
Living with spouse	143 (72.60)	54.57 ± 9.10		
Living with children	28 (14.20)	50.79 ± 4.96		
Other	9 (4.60)	54.67 ± 8.10		
Per capita monthly household income (RMB)			3.178	0.025
< 2000	9 (4.60)	60.33 ± 13.95		
2000–4999	36 (18.30)	56.14 ± 10.97		
5000–10,000	66(33.50)	54.36 ± 8.77		
>10,000	86 (43.70)	52.59 ± 6.27		
Duration of diabetes			1.758	0.175
< 5 years	99 (50.20)	53.05 ± 7.48		
5–10 years	33 (16.80)	55.7 ± 9.84		
> 10 years	65 (33.00)	55.15 ± 9.66		
Complications of diabetes			5.261	0.023
Yes	69 (35.00)	56.1 ± 9.97		
No	128 (65.00)	53.16 ± 7.76		
Comorbidity			1.258	0.263
Yes	159 (80.71)	54.44 ± 7.11		
No	38 (19.29)	53.13 ± 9.03		
Abdominal obesity			0.015	0.902
Yes	178 (9.60)	54.26 ± 8.74		
No	19 (90.40)	53.53 ± 8.41		
Hypoglycemia			14.746	< 0.001
Yes	44 (22.30)	58.11 ± 11.33		
No	153 (77.70)	53.06 ± 7.44		
Ketoacidosis			0.063	0.803
Yes	10 (5.10)	54.80 ± 10.19		
No	187 (94.90)	54.05 ± 8.61		
Patient origin			13.055	< 0.001
Outpatient	117 (59.40)	52.5 ± 7.22		
Inpatient	80 (40.60)	56.65 ± 10.02		
Primary treatment			0.323	0.809
Lifestyle	24 (12.20)	53.04 ± 6.70		
Oral hypoglycemic agents	76 (38.60)	54.07 ± 9.85		
Insulin injections	23 (11.70)	55.52 ± 8.72		
Insulin injections and oral hypoglycemic agents	74 (37.60)	54.27 ± 8.06		
GLP-1RA use			7.842	0.006
Yes	21 (10.70)	58.62 ± 11.13		
No	176 (89.30)	53.66 ± 8.23		

**Table 2:** Current status of SSCI and T2DHPS in overweight/obese patients with T2DM (*N* = 197, *x* ± *s*).

**Variables**	**Average score**	**Total score of dimensions**	**Average score of each item**
SSCI total score	54.19 ± 8.69	120	2.25 ± 0.36
Self-stigma	30.59 ± 6.01	65	2.35 ± 0.46
Enacted stigma	23.60 ± 3.44	55	2.14 ± 0.31
T2DHPS total score	87.75 ± 16.75	140	3.13 ± 0.59
Physical activity	18.35 ± 7.72	35	2.62 ± 1.10
Risk reduction	19.88 ± 5.33	35	2.61 ± 0.79
Stress management	18.68 ± 3.29	25	3.73 ± 0.65
Enjoy life	10.82 ± 2.25	15	3.85 ± 0.66
Health responsibility	9.48 ± 2.74	15	3.16 ± 0.91
Healthy diet	9.80 ± 2.78	15	3.26 ± 0.92

**Table 3:** Correlation between stigma and health-promoting behaviors in overweight/obese patients with T2DM.

**Variables**	**SSCI total score**	**Self-stigma**	**Enacted stigma**	**T2DHPS total score**	**Physical activity**	**Risk reduction**	**Stress management**	**Enjoylife**	**Health responsibility**	**Healthy diet**
SSCI total score	1									
Self-stigma	0.955⁣^∗∗^	1								
Enacted stigma	0.856⁣^∗∗^	0.665⁣^∗∗^	1							
T2DHPS total score	−0.144⁣^∗^	−0.098	−0.192⁣^∗∗^	1						
Physical activity	−0.069	−0.052	−0.082	0.822⁣^∗∗^	1					
Risk reduction	−0.011	0.034	−0.088	0.797⁣^∗∗^	0.549⁣^∗∗^	1				
Stress management	−0.312⁣^∗∗^	−0.277⁣^∗∗^	−0.305⁣^∗∗^	0.649⁣^∗∗^	0.337⁣^∗∗^	0.440⁣^∗∗^	1			
Enjoy life	−0.254⁣^∗∗^	−0.224⁣^∗∗^	−0.249⁣^∗∗^	0.645⁣^∗∗^	0.344⁣^∗∗^	0.482⁣^∗∗^	0.919⁣^∗∗^	1		
Health responsibility	−0.059	−0.007	−0.136	0.555⁣^∗∗^	0.272⁣^∗∗^	0.400⁣^∗∗^	0.274⁣^∗∗^	0.301⁣^∗∗^	1	
Healthy diet	0.004	0.043	−0.065	0.560⁣^∗∗^	0.290⁣^∗∗^	0.314⁣^∗∗^	0.314⁣^∗∗^	0.325⁣^∗∗^	0.370⁣^∗∗^	1

⁣^∗^*p* < 0.05, ⁣^∗∗^*p* < 0.01.

**Table 4:** Multiple stratified linear regression analysis of stigma in overweight/obese patients with T2DM.

**Variables**	**B**	**SE**	**β**	**t**	**p**	**95%CI**
Per capita monthly household income (RMB)	−1.250	0.636	−0.127	−1.966	0.051	−2.505~0.004
Hypoglycaemia	3.327	1.354	0.160	2.457	0.015	0.655~5.999
Patient origin	3.834	1.162	0.217	3.300	0.001	1.542~6.125
GLP-1RA use	4.213	1.804	0.150	2.335	0.021	0.654~7.773
Retinopathy	2.725	1.864	0.106	1.462	0.146	−0.953~6.403
Nephropathy	1.054	1.576	0.047	0.669	0.504	−2.056~4.164
Neuropathy	2.735	1.575	0.129	1.736	0.084	−0.373~5.842
T2DHPS total score	0.002	0.044	0.004	0.052	0.959	−0.085~0.089
Stress management	−1.105	0.430	−0.419	−2.567	0.011	−1.954~−0.256
Enjoy life	0.873	0.622	0.226	1.403	0.162	−0.355~2.101

*Note*: *F* = 7.162, *p* < 0.001, *R*^2^ = 0.278, *R*_ad_^2^ = 0.239.

## Data Availability

The data supporting our findings are available from the corresponding author upon reasonable request.
